# Multi-regression analysis revealed a relationship between l-serine and methionine, a component of one-carbon metabolism, in the normal control but not in the schizophrenia

**DOI:** 10.1186/s12991-016-0113-3

**Published:** 2016-08-31

**Authors:** Yumiko Takano, Yuji Ozeki, Masae Sekine, Kumiko Fujii, Takashi Watanabe, Hiroaki Okayasu, Takahiro Shinozaki, Akiko Aoki, Kazufumi Akiyama, Hiroshi Homma, Kazutaka Shimoda

**Affiliations:** 1 Department of Psychiatry, Dokkyo Medical University School of Medicine, 880 Kitakobayashi, Mibu, Tochigi Japan; 2 Department of Mental Disorder Research, National Institute of Neuroscience, National Center of Neurology and Psychiatry, Tokyo, Japan; 3 Laboratory of Biomolecular Science, Graduate School of Pharmaceutical Sciences, Kitasato University, Tokyo, Japan; 4 Department of Biological Psychiatry and Neuroscience, Dokkyo Medical University School of Medicine, Mibu, Tochigi Japan

**Keywords:** Schizophrenia, One-carbon metabolism (OCM), Methionine, l-serine, Homocysteine, Folate

## Abstract

**Background:**

Alterations in one-carbon metabolism (OCM) have been observed in patients with schizophrenia (SZ), but a comprehensive study of OCM has not yet been conducted. A carbon atom is transferred from l-serine to methionine during OCM, but the relationship between l-serine and methionine in SZ is not yet known. We investigated the relationship between l-serine and methionine to obtain a comprehensive understanding of OCM in SZ.

**Methods:**

We recruited forty-five patients with SZ and thirty normal controls (NC). Whole blood, plasma, and DNA specimens were obtained from all participants. Plasma l-serine, d-serine, glycine, methionine, and total homocysteine levels were measured using high-performance liquid chromatography. Plasma vitamin B12 and total folate were measured using a chemiluminescent protein-binding immunoassay. Clinical symptoms were estimated using the positive and negative syndrome scale (PANSS). The methylenetetrahydrofolate reductase (MTHFR) C667T genotype and A298C genotype, which are involved in MTHFR activity, were determined using the TaqMan genotyping assay system.

**Results:**

Analysis of variance was used to confirm that the SZ cohort has higher plasma homocysteine levels and lower plasma folate levels than the NC group. Multi-regression analysis revealed a relationship between l-serine and methionine in the NC group but not in the SZ group. The MTHFR genotype did not affect the relationship between l-serine and methionine in each group. The total PANSS score was significantly related to d-serine and folate levels and to age. Positive PANSS scores were significantly related to both glycine and sex. In addition, both glycine and d-serine were significantly correlated with negative PANSS scores.

**Conclusions:**

We found impairment of the relationship between l-serine and methionine in SZ. Clinical symptoms of SZ were partially correlated with the OCM components. These findings contributed to our understanding of OCM alteration in SZ and may explain why the alteration occurs.

## Background

**Fig. 1:**
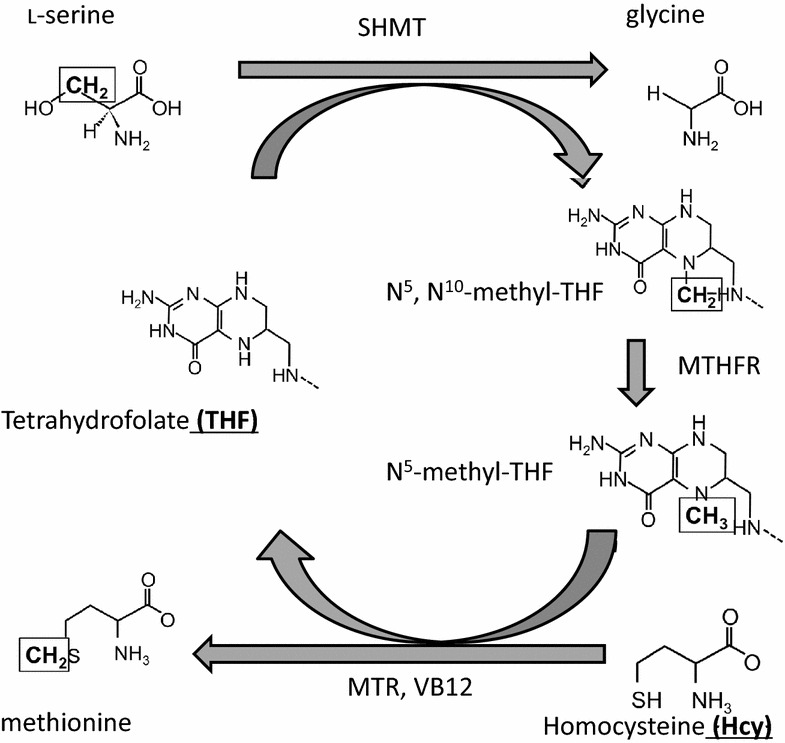
The carbon atom of one-carbon metabolism is exchanged between l-serine and methionine. The folate cycle obtains the methylene group (one carbon atom) from l-serine via the catalysis of serine hydroxymethyltransferase (SHMT) and subsequently donates the carbon to methionine synthesis. l-serine becomes glycine by offering the methylene group to methionine synthesis. Methionine is synthesized from homocysteine (Hcy) using a folate cycle-derived carbon atom. *MTHFR* methylenetetrahydrofolate reductase; *MTR* 5-methyltetrahydrofolate-homocysteine methyltransferase

One-carbon metabolism (OCM) alteration has been observed in patients with schizophrenia (SZ) [1–4]. OCM involves methionine cycles and folate cycles to generate diverse outputs such as substrates for methylation reactions and the maintenance of redox status [5]. The folate cycle results in the transfer of the methylene group (one carbon atom) from l-serine by catalysis of serine hydroxymethyltransferase to the methionine cycle. l-serine is converted into glycine by donating its methylene group to the methionine cycle, which is a part of OCM. Methionine is synthesized from homocysteine (Hcy) using a carbon atom that is obtained from the folate cycle (Fig. 1) [5]. Some components of OCM are altered in patients with SZ. Elevated Hcy [4, 6–10] and low folate [11, 12] and vitamin B12 (VB12) levels [13] have been reported in patients with SZ; however, the relationship between each alteration is unknown. For example, the peripheral serine level is considered to be significantly higher in SZ patients than in normal controls (NC), especially in male controls [14, 15]. Yet, l-serine’s relationship with OCM has not been well investigated. The carbon atom that belongs to OCM is exchanged between l-serine and methionine. Therefore, we explored the relationship between methionine and l-serine to obtain a comprehensive understanding of the role of OCM in the etiology of SZ.

## Methods

### Participants

We recruited forty-five SZ and thirty NC patients between 2010 and 2013. All participants were treated at one of the three hospitals: Dokkyo Medical University Hospital, Mori Hospital, or Shimotsuga General Hospital. Demographic data of the participants are shown in Table 1. All SZ patients were in a stable state. Participants with severe complications that could be confirmed by physical examination and laboratory testing for liver function, renal function, lipid metabolism and glucose metabolism, were excluded. The results of physical examinations and laboratory testing of patients were obtained from medical records by research psychiatrists (YT, YO, KF, TW, HO, TS, AA, KA, and KS). NC participants were interviewed by a research psychiatrist (KA) to obtain detailed medical histories and confirm that they were not suffering from mental illness or severe physical disease. Psychiatric histories were taken from each medical record by a research psychiatrist (KA). Two independent psychiatrists used the American Psychiatric Association’s Diagnostic and Statistical Manual of Mental Disorders [16] criteria to assess all patients (YO and KA). The positive and negative syndrome scale (PANSS) [17] was used to measure clinical symptoms in all patients by a research psychiatrist who was trained and experienced in the rating scales (KA). After fully explaining the study procedures, we obtained written, informed consent from all participants. The study was approved by the ethics committee of the Dokkyo Medical University School of Medicine.

**Table 1:** Demographics of participants

	Schizophrenia	Healthy control
Number of participant	45	30
Sex	Male: 27	Male: 18
Age	50.6 ± 12.6	45.5 ± 13.0
Number of smoker	15	8
Duration of illness (years), mean ± SD	27.3 ± 12.8	
CP equivalent dose (mg/day), mean ± SD	667.58 ± 635.28	
l-serine (μM), mean ± SD	91.2 ± 21.1	85.6 ± 22.4
d-serine (μM), mean ± SD	1.50 ± 0.42	1.37 ± 0.45
Glycine (μM), mean ± SD	194.9 ± 64.0	176.5 ± 49.6
Vitamin B12 (pg/mL), mean ± SD	348.7 ± 132.6	309.5 ± 154.0
Folate (ng/mL), mean ± SD*	4.4 ± 1.7	5.7 ± 2.7
Homocysteine (μM), mean ± SD**	13.4 ± 8.3	9.1 ± 2.6
Methionine (μM), mean ± SD	13.1 ± 5.3	11.2 ± 7.1
PANSS score, mean ± SD	72.7 ± 17.9	
Positive, mean ± SD	14.4 ± 5.4	
Negative, mean ± SD	23.0 ± 6.0	

Positive: subscale of positive syndrome scale in PANSS

Negative: subscale of negative syndrome scale in PANSS

*CP* chlorpromazine, *SD* standard deviation, *PANSS* positive and negative syndrome scale

* *p* < 0.05, ** *p* < 0.01

### Plasma l-serine, d-serine, glycine, methionine, and total homocysteine analysis

Before noon, 8 mL whole blood samples were drawn from each participant and placed into BD Vacutainer^®^ CPT™ cell preparation tubes containing sodium heparin (Becton, Dickinson Co., Plainfield, NJ, USA). We followed the manufacturer’s instructions to separate plasma from the whole blood sample, which was then stored at −80 °C.

The samples for high-performance liquid chromatography were prepared as previously described [18]. Briefly, 150 μL of H_2_O and 10 μL of 40 % (w/v) trichloroacetic acid were added to 50 μL of plasma and mixed by vortexing. After centrifugation at 15,000 rpm for 10 minutes at 4 °C, 150 μL of the supernatant was removed and added to 130 μL of H_2_O, 100 μL of 200 mM borate buffer (pH 8.5), and 20 μL of 1 M NaOH before being vortex-mixed. Fluorescent derivatization was conducted by adding 40 μL of 50 mM borate buffer (pH 8.5) and 50 μL of 10 mM 4-fluoro-7-nitro-benzoxadiazole (NBD-F) solution to 20 μL of the above-described solution. The mixture was incubated at 60 °C for 5 minutes. The reaction was stopped by adding 890 μL of 2 % trifluoroacetic acid (TFA).

Separation and fluorometric detection of NBD-glycine and NBD-methionine were conducted according to a previous study [19], except that TSKgel ODS-80Ts QA (250 × 4.6 mm, i.d., 5 μm, Tosoh Corporation, Tokyo, Japan) was used as an ODS column, and the elution solvent and program were modified accordingly.

Separation and fluorometric detection of NBD-d- and -l-serine were performed according to the methods of a previous study [18], except that InertSustain (250 × 4.6 mm, i.d., 5 μm, GL Sciences Inc., Tokyo, Japan) and Sumichiral OA-3200 (250 × 4.6 mm, i.d., 5 μm, Sumika Analytical Center, Osaka, Japan) were used as a reverse-phase octyl silica column and a Pirkle-type chiral column, respectively. Additionally, separation on the octyl silica column and the chiral column was conducted using mobile phases of 1 % methanol and 0.01 % TFA in 15 % acetonitrile (0.8 mL/min) and 1 mM citrate in methanol: acetonitrile (95:5; 1.0 mL/min), respectively.

Hcy was labeled with 4-fluoro-7-sulfamoylbenzofurazan and detected using a fluorescence detector, according to the method of a previous study [20]. The chemicals were purchased from Sigma-Aldrich Japan (Tokyo, Japan), except as indicated above.

### Plasma VB12 and total folate analysis

VB12 and total folate were measured using a chemiluminescent protein-binding immunoassay (Beckman Coulter, CA, USA). All assays were performed according to the manufacturers’ instructions.

### Genotyping

The *MTHFR* C677T genotype (rs 1801133) and A1298C (rs 1801131) genotype, in which minor alleles elevate the Hcy concentration [21], were determined using a commercially available TaqMan single nucleotide polymorphism Genotyping Assay Kit (Applied Biosystems, Life Technologies, CA, USA) and a stratagene Mx3000P QPCR system (Agilent Technologies, CA, USA), according to the protocol recommended by the manufacturer. The assay IDs were C_1202883 and C_850486, respectively.

### Statistical analysis

We used analysis of covariance to compare the demographics data and measurement results (plasma concentration of l-serine, d-serine, glycine, VB12, folate, Hcy, and methionine) of SZ and NC. Allelic and genotypic frequencies of the *MTHFR* C677T and A1298C polymorphisms in SZ and NC, respectively, were compared using the χ^2^ test. Multiple linear regression analysis, with both the forced entry model and the backward elimination model, was used to examine the relationship between the plasma methionine level and plasma amino acids (l-serine, d-serine, and glycine), Hcy, VB12, and folate concentrations in both SZ and NC. In the NC group, l-serine, d-serine, glycine, Hcy, VB12 and folate levels, age, sex, and smoking status were considered independent variables. In the SZ group, l-serine, d-serine, glycine, Hcy, VB12 and folate levels, age, sex, antipsychotic dose (chlorpromazine-equivalent dose), duration of disease, and smoking status were considered as independent variables. Furthermore, the relationship between the PANSS score (total score, positive score, and negative score) and data (demographic and measured) were examined in SZ patients using multiple linear regression analysis with both the forced entry model and the backward elimination model. Multicollinearity in each model was estimated using a variance inflation factor. A variance inflation factor of greater than five is generally considered evidence of multicollinearity [22]. All statistical analyses were performed using the statistical package PASW, Version 18.0 (SPSS Japan Inc., Tokyo, Japan). Statistical significance was defined as a p-value of less than 0.05.

## Results

The mean Hcy level was significantly higher in the SZ group than in the NC group (*F* = 7.58; *p* = 0.007; Table 1), whereas the mean folate level was significantly lower in the SZ group than in the NC group (*F* = 6.23; *p* = 0.015; Table 1). With regard to genotype, no significant differences were found between the two groups (rs 180131: χ^2^ = 3.26, *p* = 0.20; rs 180133: χ^2^ = 0.97, *p* = 0.62; Table 2).

**Tabel 2:** Allele frequency and genotyping of the MTHFR SNPs in participants

	Allele	Genotype	*p* value for HWE
rs1801131			
Schizophrenia	A: 76, C: 14	AA: 34, AC: 8, CC: 3	0.20
Control	A: 51, C: 9	AA: 21, AC: 9, CC: 0	
rs1801133			
Schizophrenia	C: 52, T: 38	CC: 17, CT: 18, TT: 10	0.62
Control	C: 38, T: 22	CC: 12, CT: 14, TT: 4	

*MTHFR* methylenetetrahydrofolate reductase, *HWE* Hardy–Weinberg equilibrium

Multiple regression analysis was performed to determine the relationship between methionine and other factors. In the NC group, multiple regression analysis indicated no reliance in the forced entry model [adjusted *R*^2^ = 0.16, *F* (9, 20) = 1.51; *p* = 0.21]; however, reliance was obtained in the backward elimination model (adjusted *R*^2^ = 0.41, *F* (2, 27) = 7.76; *p* = 0.001). In this model, l-serine level (*t* = 2.8; β = 0.43 ± 0.15; *p* = 0.008), age (*t* = 2.62; β = 0.53 ± 0.15; *p* = 0.001), and sex (*t* = −2.15; β = −0.33 ± 0.15; *p* = 0.041) were significantly correlated with the methionine level. In the SZ group, no factor was associated with the methionine level in both the forced entry model and the backward elimination model (Table 3) (forced entry model: adjusted *R*^2^ = 0.12, *F* (13, 31) = 0.67, *p* = 0.78; backward elimination model: adjusted *R*^2^ = 0.06, *F* (2, 42) = 3.33, *p* = 0.76).

**Table 3:** Relationship between methionine and other factors in patients with schizophrenia and normal controls: multiple regression analysis

	Forced entry model			Backward elimination model		
	β ± SD	*p* value	VIF	β ± SD	*p* value	VIF
Normal control						
Sex	−0.33 ± 0.23	0.17	1.90	−0.33 ± 0.15	0.04	1.18
Age	0.55 ± 0.20	0.01	1.51	0.53 ± 0.15	<0.001	1.09
Smoking status	−0.05 ± 0.21	0.92	1.55			
Vitamin B12	−0.11 ± 0.28	0.70	2.76			
Folate	−0.08 ± 0.24	0.75	2.06			
Homocysteine	−0.08 ± 0.21	0.71	1.55			
l-serine	0.40 ± 0.28	0.17	2.79	0.43 ± 0.15	0.008	1.13
d-serine	−0.03 ± 0.29	0.92	3.06			
Glycine	0.02 ± 0.28	0.94	1.91			
Genotype131	−0.03 ± 0.19	0.89	1.25			
Genotype133	0.08 ± 0.25	0.75	2.37			
Patients with schizophrenia						
Sex	−0.06 ± 0.28	0.83	2.71			
Age	0.21 ± 0.37	0.57	4.76			
Duration of illness	−0.02 ± 0.32	0.95	3.32			
Smoking status	0.12 ± 0.25	0.63	2.19			
CP equivalent dose	−0.01 ± 0.28	0.95	1.53			
Vitamin B12	0.06 ± 0.22	0.74	1.29			
Folate	−0.11 ± 0.21	0.61	1.64			
Homocysteine	−0.29 ± 0.23	0.23	1.93	−0.35 ± 0.16	0.14	1.18
l-serine	0.15 ± 0.26	0.55	2.31			
d-serine	−0.13 ± 0.27	0.63	2.58			
Glycine	0.10 ± 0.26	0.67	2.05			
Genotype131	0.32 ± 0.24	0.20	2.07	0.35 ± 0.16	0.14	1.36
Genotype133	0.21 ± 0.28	0.46	2.77	0.27 ± 0.18	0.14	1.18

*β*  standardised partial regression coefficient, *VIF* variance inflation factor, *CP* chlorpromazine, *SD* standard deviation

The factors that related to the PANSS score were determined using multiple regression analysis in SZ patients. The forced entry model of the total PANSS score indicated no reliance [adjusted *R*^2^ = 0.26, *F* (13, 31) = 1.94; *p* = 0.077]; however, the backward elimination model was reliant [adjusted *R*^2^ = 0.32, *F* (5, 39) = 3.92; *p* = 0.005]. In this model, levels of folate (*t* = 2.18; β = 0.35 ± 0.16; *p* = 0.037), d-serine (*t* = 2.68; β = 0.38 ± 0.16; *p* = 0.012), and age (*t* = 0.37; β = 0.54 ± 0.15; *p* < 0.001) were indicated as factors that were related to the total PANSS score. No reliance was observed in the forced entry model for either a positive PANSS score [adjusted *R*^2^ = 0.33, *F* (13, 31) = 2.32; *p* = 0.035] or a negative PANSS score [adjusted *R*^2^ = 0.17, *F* (13, 31) = 1.54; *p* = 0.17]. In contrast, the backward elimination model was reliable in both positive [adjusted *R*^2^ = 0.30, *F* (6, 38) = 9.04; *p* = 0.001] and negative PANSS scores [adjusted *R*^2^ = 0.17, *F* (6, 38) = 3.58; *p* = 0.024]. Positive PANSS scores were significantly related to both the glycine level (*t* = 5.50; β = 0.47 ± 0.13; *p* = 0.001) and sex (*t* = −3.69; β = −0.71 ± 0.19; *p* < 0.001). In addition, both glycine (*t* = −3.16; β = −0.61 ± 0.16; *p* = 0.004) and d-serine (*t* = 2.44; β = 0.47 ± 0.19; *p* = 0.021) were significantly correlated with negative PANSS scores (Table 4).

**Table 4:** Relationship between PANSS score and other factors in patients with schizophrenia: multiple regression analysis

	Forced entry model			Backward elimination model		
	β ± SD	*p* value	VIF	β ± SD	*p* value	VIF
PANSS score						
Sex	−0.13 ± 0.22	0.57	2.42			
Age	−0.62 ± 0.32	0.06	5.04	−0.54 ± 0.15	<0.001	1.16
Smoking status	−0.15 ± 0.20	0.44	1.91			
Duration of illness	0.16 ± 0.25	0.53	3.22			
CP equivalent dose	0.11 ± 0.18	0.52	1.38			
Vitamin B12	0.05 ± 0.15	0.76	1.31			
Folate	0.30 ± 0.19	0.12	1.73	0.35 ± 0.16	0.04	1.41
Homocysteine	0.38 ± 0.21	0.09	2.31	0.27 ± 0.16	0.10	1.43
l-serine	0.00 ± 0.00	1.00	3.26			
d-serine	0.43 ± 0.25	0.10	3.08	0.38 ± 0.14	0.01	1.07
Glycine	−0.13 ± 0.20	0.53	2.13			
Methionine	0.34 ± 0.16	0.05	1.35	0.29 ± 0.15	0.07	1.29
Genotype131	−0.29 ± 0.20	0.16	1.96	−0.25 ± 0.15	0.10	1.18
Genotype133	−0.14 ± 0.23	0.55	2.61			
Positive PANSS score						
Sex	−0.03 ± 0.21	0.89	2.42	−0.71 ± 0.19	<0.001	2.21
Age	−0.68 ± 0.30	0.04	5.04			
Smoking status	−0.04 ± 0.18	0.82	1.91			
Duration of illness	0.25 ± 0.24	0.32	3.22	0.31 ± 0.19	0.11	2.07
CP equivalent dose	0.12 ± 0.12	0.45	1.38			
Vitamin B12	0.14 ± 0.16	0.38	1.31			
Folate	0.32 ± 0.18	0.08	1.73	0.25 ± 0.14	0.08	1.12
Homocysteine	0.31 ± 0.20	0.15	2.31			
l-serine	0.07 ± 0.24	0.77	3.26			
d-serine	0.22 ± 0.24	0.36	3.08			
Glycine	0.31 ± 0.20	0.13	2.13	0.47 ± 0.13	<0.001	1.06
Methionine	0.26 ± 0.16	0.11	1.35	0.19 ± 0.15	0.20	1.25
Genotype131	−0.32 ± 0.19	0.11	1.96	−0.23 ± 0.14	0.11	1.16
Genotype133	−0.06 ± 0.22	0.78	2.61			
Negative PANSS score						
Sex	−0.05 ± 0.23	0.82	2.42			
Age	−0.42 ± 0.34	0.23	5.04	−0.25 ± 0.15	0.11	1.07
Smoking status	−0.18 ± 0.21	0.38	1.91	−0.27 ± 0.16	0.11	1.27
Duration of illness	0.15 ± 0.27	0.58	3.22			
CP equivalent dose	0.12 ± 0.24	0.50	1.38			
Vitamin B12	0.05 ± 0.18	0.79	1.31			
Folate	0.14 ± 0.20	0.49	1.73			
Homocysteine	0.37 ± 0.23	0.12	2.31	0.24 ± 0.18	0.19	1.48
l-serine	0.14 ± 0.27	0.61	3.26			
d-serine	0.30 ± 0.26	0.27	3.08	0.47 ± 0.19	0.02	1.73
Glycine	−0.53 ± 0.22	0.02	2.13	−0.61 ± 0.20	0.004	1.76
Methionine	0.28 ± 0.17	0.12	1.35			
Genotype131	−0.37 ± 0.21	0.09	1.96	−0.16 ± 0.15	0.30	1.10
Genotype133	−0.31 ± 0.24	0.22	2.61			

*β* standardised partial regression coefficient, *VIF* variance inflation factor

## Discussion

In this study, we found a relationship between methionine and l-serine in the NC group, but this relationship did not exist in the SZ group. Increased plasma Hcy levels and decreased folate levels were observed in the SZ group. The PANSS scores of clinical symptoms correlated with folate, d-serine, and glycine levels.

A number of studies have reported OCM alteration in SZ [1–4]. In the OCM cycle, methionine is synthesized by a chemical reaction between Hcy and a carbon atom provided by l-serine. Elevated peripheral l-serine levels have also been reported in SZ [14, 15]. Although l-serine and methionine exchange a carbon atom in OCM, the relationship between l-serine and methionine in OCM has not been investigated in SZ. For this reason, we investigated the relationship between l-serine and methionine in schizophrenia. In this investigation, we confirmed the relationship between methionine and l-serine in the NC group; however, no relationship was identified in SZ. This result indicates an impairment of the relationship between l-serine and methionine in SZ and that the impairment may induce high peripheral Hcy levels and high peripheral l-serine levels in SZ. In agreement with previous studies [4, 6–10], we confirmed that the peripheral Hcy level was higher in the SZ group than in the NC group. High peripheral l-serine levels in SZ were also reported [14, 15] but have not been confirmed because this observation was made in a small number of participants. To date, the reason why the peripheral Hcy level and l-serine level is higher in SZ than in NC remains unknown.

Low folate levels [11] and low VB12 levels [12, 13] have also been reported in SZ. We confirmed low folate but not low VB12 levels in the present study. Folate carries a carbon atom from l-serine to methionine, which is synthesized from Hcy (Fig. 1). MTHFR plays an important role in this pathway because MTHFR catalyzes the conversion of 5,10-methylenetetrahydrofolate to 5-methyltetrahydrofolate, a co-substrate for Hcy remethylation to methionine. Some allelic variants change enzymatic activity. For example, the *MTHFR* C677T and A1298C genotypes reduce the activity of MTHFR and finally elevate the peripheral Hcy level [23]. These allelic variants have been indicated as risk factors of SZ [24–31]. Therefore, the disruption of the relationship between l-serine and methionine could be elucidated by the frequency of MTHFR allele variants. Nevertheless, we found no relationship between the allele variant frequency and the Hcy level (data not shown), even though a higher level of Hcy in SZ was observed. Because the effect size of MTHFR allelic variants for the plasma Hcy levels was relatively low (odds ratio: 1.14) [9], we cannot conclusively indicate the allelic variant effect as a reason for impairment of the relationship between l-serine and methionine. A study with an increased number of participants would be needed to study this further.

Although we found no effect of the MTHFR allele variant, the relationship between l-serine and methionine was altered in SZ, suggesting another reason for this alteration. For example, the low supply or high consumption of folate could be a contributing factor to the disruption of plasma folate levels, which were lower in the SZ group than in the NC group. Further studies and confirmation of these findings are necessary to determine the reason for our finding of an altered relationship between l-serine and methionine in SZ. It is worth noting; however, that MTHFR allelic variants may not be the only cause of OCM alteration in SZ.

With regard to the relationship between OCM and clinical symptoms of SZ, significant negative relationships between the serum folate levels and the total PANSS scores and the negative PANSS symptom score in SZ [32] have been reported. Additionally, there was a positive relationship between the serum Hcy levels and the total PANSS scores [32]; however, these relationships were not confirmed in our results. We found a positive relationship between plasma folate and total PANSS score. The Hcy levels did not correlate with clinical symptoms in our study. No firm conclusion can be made concerning a relationship between clinical symptoms and peripheral levels of folate or Hcy in SZ.

Calcia et al., found that d-serine levels were inversely correlated with the total Brief Psychiatric Rating Scale (BPRS) score and with the BPRS score for negative symptoms, but not with the BPRS score for positive symptoms [33]. In contrast, Hone et al., reported that d-serine serum levels were not associated with the PANSS or Scale for the Assessment of Negative Symptoms (SANS) total and subscales scores [34]. They also showed serum glycine levels to be negatively associated with the intensity of negative symptoms assessed by the PANSS negative subscale [35]. In our study, the plasma d-serine concentration was positively correlated with both the total PANSS score and the positive PANSS score. We also found the plasma glycine levels to be negatively correlated with a negative PANSS score and positively correlated with a positive PANSS score. These findings were statistically significant. Further evaluation of the correlation between clinical symptoms and peripheral d-serine or glycine levels is necessary to explain the discrepancies between studies. Because both d-serine and glycine are considered adjunctive agents [36–38], alteration of both of these amino acids is likely to be found in SZ. Nevertheless, no firm conclusion has been reached concerning the relationship between clinical symptoms and peripheral levels of d-serine or glycine.

One limitation of our study was the relatively low number of participants. This resulted in a low allele variant effect on MTHFR enzyme activity, making it difficult to evaluate the effect of the allelic variant in our findings. The other limitation was that all SZ patients were receiving antipsychotic medication, which could have impacted our results. Even though no statistical effect was observed, we cannot completely rule out the effect of antipsychotic medications on OCM. Until now, the effect of antipsychotics medications on OCM was mainly focused on the relationship between antipsychotics and Hcy. However, the results are inconsistent. Nishi et al., did not find a relationship between antipsychotic dose and plasma Hcy level [9]. In contrast, Misaki et al., found an increase of serum Hcy level by second-generation antipsychotic medication [39]. They did not examine the peripheral methionine level. The inconsistencies make it difficult to determine the effect that antipsychotic medication may have on OCM, which should be examined in future studies. A larger sample size would allow patients to be divided into subgroups by medication to gain a better understanding of the effect of antipsychotic medication and provide results applicable to clinical situations. Furthermore, the cross-sectional nature of this study design did not allow a causal relationship to be determined between severity of symptoms and levels of folate, d-serine, and glycine.

## Conclusions

In conclusion, we found no relationship between l-serine and methionine in SZ. It is our hope that the findings of this study will contribute to a comprehensive understanding of OCM alteration in SZ. Determining the cause of OCM alterations could lead to investigations of new therapeutic targets in future studies.
